# Supplementary material for: mRNA-Loaded Lipid Nanoparticles Targeting Immune Cells in the Spleen for Use as Cancer Vaccines
Source: Pharmaceuticals (Basel). 2022 Aug 18;15(8):1017. doi: 10.3390/ph15081017 (PMC9415712; doi:10.3390/ph15081017)
Supplement: Supplementary file 1 [file pharmaceuticals-15-01017-s001.zip › pharmaceuticals-1868567-supplementary.pdf]

pharmaceuticals

MDPI

Research Article

mRNA-loaded lipid nanoparticles targeting immune cells in the spleen for use as cancer vaccines

Ryoya Shimosakai[1], Ikramy A. Khalil[1,2,*], Seigo Kimura[1], and Hideyoshi Harashima[1,3,*]

Supplementary Table S1. Characterization of different LNPs and RNA-LPX

A. Characterization of DODAP-LNPs prepared with different ratios of DODAP and Cholesterol ratios[1]

DODAP/Chol	Size (nm)	PDI	ζ-potential (mV)	EE (%)
28.5/20	120±12	0.106±0.012	-5.04±8	85.4±16
38.5/10	118±16	0.121±0.013	-4.7±8	85.7±20
48.5/0	123±10	0.085±0.033	-10±8	78.3±14

[1] Values are mean ± SD of at least three different preparations

B. Characterization of DODAP-LNPs prepared with different ratios of DODAP and DOPE ratios[1]

DODAP/DOPE	Size (nm)	PDI	ζ-potential (mV)	EE (%)
60/28.5	128±15	0.115±0.024	-9.5±6	86.9±16
70/18.5	126±25	0.138±0.053	-11.2±7	90.7±20
80/8.5	146±6	0.101±0.022	-16.3±10	96.6±14

[1] Values are mean ± SD of at least three different preparations

C. Characterization of DODAP-LNPs prepared with different amounts of total lipids[1]

Lipid (nmol)	Size (nm)	PDI	ζ-potential (mV)	EE (%)
160	132±7	0.135±0.046	-17±2	73±1
240	119±10	0.145±0.041	-11.1±10	79.8±12
480	129±18	0.100±0.052	-11±2	85.5±12

[1] Values are mean ± SD of at least three different preparations

D. Characterization of RNA lipoplexes[1]

	Size (nm)	PDI	ζ-potential (mV)
RNA-LPX	298±44	0.335±0.04	-40.5±14

[1] Values are mean ± SD of three different preparations

Supplementary Figure S1. Flow cytometry analysis of cellular uptake in splenocytes

A.

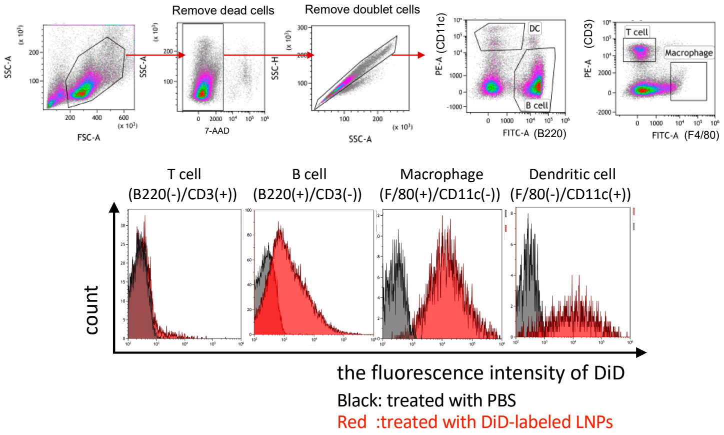

B.

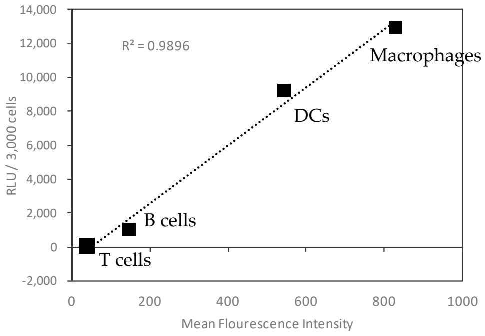

A. Gating strategy and histogram. In the histogram, red: DiD labeled LNP, black: PBS.

B. The correlation between gene expression per cell and cellular uptake per cell.
